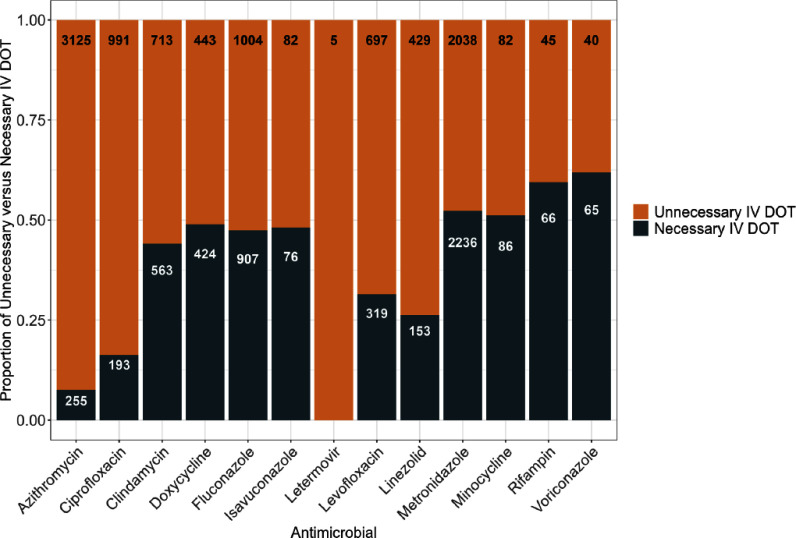# Estimated Carbon Dioxide Emissions Associated with Unnecessary Intravenous Antimicrobials Administered in the Hospital Setting

**DOI:** 10.1017/ash.2025.251

**Published:** 2025-09-24

**Authors:** Anil Chakravorty, Nicholas Newman, Courtney Veltri, Emily Spivak, Michelle Hecker, Leila Hojat

**Affiliations:** 1 University Hospitals Cleveland Medical Center; 2 University Hospitals Cleveland Medical Center; 3 University of Utah School of Medicine and Salt Lake City VA; 4 MetroHealth Medical Center; 5 Emory University Hospital Midtown

## Abstract

**Background:** Hospitals have been recognized as major drivers of the deleterious environmental impacts of human industry. Intravenous (IV) therapy and its associated preparation and administration materials account for a large component of the plastic waste produced by hospitals. Switch therapy refers to transitioning antimicrobials from the IV to the enteral (PO) route. Despite this being a well-established practice, it has not been studied extensively in the context of reducing hospital-generated plastic waste. This study investigated the waste which could be avoided through optimization of IV to PO switch therapy. **Method:** A retrospective cohort study was performed at a large academic center in a metropolitan area. We included all adult patients receiving an IV antimicrobial with a highly bioavailable PO equivalent between October 2023 and September 2024. For a randomly selected subset of each agent we determined the total number of days during which patients would have been eligible for PO conversion based on our institution’s policy. This was used to determine the mean potential IV days of therapy (DOT) saved for each agent. The mean IV DOT saved were then extrapolated to the total number of patients receiving the corresponding antimicrobial agent over the course of the year to calculate a final estimated annual IV DOT which could be saved through optimized IV to PO switch therapy. A carbon emissions estimation tool was then used to estimate the carbon dioxide equivalents of the solid waste generated from the IV DOT saved. **Result:** A total of 15,037 DOT of IV antimicrobials with a highly bioavailable PO alternative were administered over the course of a year, of which an estimated 9,694 (64%) IV DOT could have been saved had appropriate switch therapy been implemented (Figure). This amounts to 2,049 kilograms of solid waste, or 0.353 metric tons of CO2 equivalents, generated through unnecessary administration of IV antimicrobials. This is equivalent to 904 miles driven, 40 gallons of gasoline consumed, 389 pounds of coal burned, or the energy required to maintain 23,392 fully depleted phone batteries at full charge throughout one day. **Conclusion:** Optimizing the implementation of IV to PO antimicrobial therapy can be an effective way of decreasing a hospital’s impact on the environment through reduction of solid waste generation. Future work should prioritize implementing life cycle assessments to broaden our understanding of how the use and production of IV medications impact the environment.

**Figure:**